# Altered platelet proteome in lupus anticoagulant (LA)-positive patients—protein disulfide isomerase and NETosis as new players in LA-related thrombosis

**DOI:** 10.1038/s12276-019-0358-4

**Published:** 2020-01-20

**Authors:** Lena Hell, Kristina Lurger, Lisa-Marie Mauracher, Ella Grilz, Christina Maria Reumiller, Georg Johannes Schmidt, Huriye Ercan, Silvia Koder, Alice Assinger, José Basilio, Johanna Gebhart, Cihan Ay, Ingrid Pabinger, Maria Zellner

**Affiliations:** 1 Clinical Division of Haematology and Haemostaseology, Department of Medicine I, Medical University of Vienna, Vienna, Austria; 2 Center for Physiology and Pharmacology, Medical University of Vienna, Vienna, Austria; 3 Department of Clinical Pharmacology, Medical University of Vienna, Vienna, Austria

**Keywords:** Protein-protein interaction networks, Predictive markers, Proteomic analysis

## Abstract

Patients with antiphospholipid syndrome (APS) are at high risk of developing venous and arterial thromboembolism (TE). The role of platelets in the pathogenesis of these prothrombotic conditions is not yet fully understood. The aim of this study was to gain mechanistic insights into the role of platelets in APS by comparing the platelet proteome between lupus anticoagulant (LA)-positive patients with (LA+ TE+) and without a history of TE (LA+ TE−) and healthy controls. The platelet proteome of 47 patients with LA, 31 with a history of TE and 16 without thrombotic history, and 47 healthy controls was analyzed by two-dimensional differential in-gel electrophoresis and mass spectrometry to identify disease-related proteins. Afterward, selected LA-related platelet proteins were validated by western blot and ELISA. Alterations of 25 proteins were observed between the study groups. STRING pathway analysis showed that LA-related protein profiles were involved in platelet activation, aggregation, and degranulation. For example, protein disulfide isomerase family members, enzymes that promote thrombosis, were upregulated in platelets and plasma of LA+ TE+ patients. Leukocyte elastase inhibitor (SERPINB1), an antagonist of neutrophil extracellular trap (NET) formation, was decreased in platelets of LA+ TE+ patients compared to healthy controls. Additionally, citrullinated histone H3, a NET-specific marker, was increased in plasma of LA+ TE+ patients. These findings suggest that decreased platelet SERPINB1 levels favor prothrombotic NETosis, especially in LA+ TE+ patients. Our findings reveal protein abundance changes connected to altered platelet function in LA-positive patients, thus suggesting a pathogenic role of platelets in thrombotic complications in APS.

## Blood coagulation: Protein changes in platelets linked to thrombosis

People at high risk for thrombosis (formation of a blood clot inside a blood vessel) show alterations in their platelet protein content. Lena Hell, Maria Zellner, Ingrid Pabinger and colleagues at the Medical University of Vienna, Austria, analyzed proteins found in blood platelets from people who were tested positive for lupus anticoagulants (LAs), antibodies known to increase the risk of thrombosis. Many proteins involved in platelet activation and blood coagulation were found at significantly different levels in people with both LAs and a history of thrombosis compared to control groups. Among these proteins were enzymes that promote clot formation, and regulators of neutrophils, white blood cells involved in the coagulation process. The findings could aid in the search for alternatives to existing anticoagulant therapies.

## Introduction

Antiphospholipid antibody syndrome (APS) is a systemic autoimmune disorder defined by the presence of antiphospholipid antibodies (aPLAs) in combination with the occurrence of venous and/or arterial thromboembolism (TE) and/or pregnancy morbidity^1^. Antiphospholipid antibodies, including anti-cardiolipin antibodies (aCLs), anti-ß2-glycoprotein I antibodies (anti-ß2GPI) and lupus anticoagulant (LA), are a heterogeneous group of autoantibodies directed toward anionic phospholipids, phospholipid-binding plasma proteins and phospholipid–protein complexes^2^ and are measured either by in vitro clotting assays, as LA interferes with phospholipid-dependent coagulation assays, or by standardized immunoassays (aCLs and anti-ß2GPI)^3^. Catastrophic APS (CAPS), characterized by rapid development of microthrombi and life-threatening multi-organ failure, is the most severe form of APS^4^.

Among all aPLAs, LA entails the highest risk of developing thrombosis^5^. Nevertheless, not all individuals with LA develop TE^6^. Several other risk factors promoting thrombosis have been identified^7,8^. However, more specific biomarkers are needed to predict and treat individual thrombotic risk.

The role of platelets in the pathogenesis of thrombosis in LA-positive patients is of particular interest. Previous studies have shown that LA is able to bind phospholipid/plasma protein complexes on platelets, thus influencing their activation and aggregation status^9–11^. LA-positive individuals show glycoprotein IIb/IIIa complex activation and upregulated CD63 expression on platelets, as well as elevated levels of soluble P-selectin^10^ and platelet-derived extracellular vesicles (EVs)^9^ in plasma. This strongly suggests that platelets play a role in the pathogenesis of thrombosis in LA-positive patients^12^.

As platelets are anucleated cells with a limited amount of mRNA, proteomics is an appropriate tool to characterize their phenotype^13^. To date, no systematic analysis of the platelet proteome of LA-positive patients in relation to their thrombotic phenotype has been conducted.

Therefore, we compared the platelet proteome of LA-positive individuals with a history of TE (LA+ TE+) to that of LA-positive individuals without a history of TE (LA+ TE−) and healthy controls using fluorescence two-dimensional differential in-gel electrophoresis (2D-DIGE), a highly accurate proteomics method to quantify and characterize disease-related protein profiles^14,15^.

## Materials and methods

### Study population

For the present study, patients from the Vienna Lupus Anticoagulant and Thrombosis Study were included. The Vienna Lupus Anticoagulant and Thrombosis Study is an ongoing, prospective, single-center cohort study enrolling adult patients persistently positive for LA (confirmed 12 weeks apart). Details on the study design have been elaborately described before^7,16^. In the current analysis, patients with a history of TE and patients without a history of TE who attended their regular follow-up visits between 2015 and 2017 were asked for new blood samples. One patient suffered from CAPS with an acute thromboembolic event at the time of study inclusion. Each patient completed a questionnaire on the medical history, and patients underwent a laboratory assessment including the determination of aPLAs. Healthy controls were individuals without APS or a history of TE from the same geographic region and ethnic background. The ethics committee of the Medical University of Vienna in accordance with the Declaration of Helsinki approved the conduct of the study (EC no. 068/2001 and 1268/2014), and each patient provided written informed consent. A summary of the study populations can be found in Table 1.

**Table 1:** Baseline demographic, clinical and laboratory data of the study cohort.

Characteristics	LA+ TE+ (*n* = 31)	LA+ TE− (*n* = 16)	Controls (*n* = 47)
Median age at study entry, y (IQR)	45 (36–52)	50 (35–68.5)	48 (31–56)
Female, *n* (%)	24 (77.4)	12 (75)	36 (78.7)
Smoking, *n* (%)	6 (19.4)	4 (25)	5 (10.6)
*Median laboratory values (IQR)*			
Platelet count × 10^9^ /L	226 (190–252)	206 (124–250)	262 (213–284)
MPV (xL fl)	10.4 (9.6–10.9)	10.6 (10.3–11.3)	10.5 (9.9–11.6)
Neutrophil count (%)	62 (58–68)	62.5 (56–69)	58.5 (54.5–64)
*History of TE, n (%)*	31 (100)	0 (0)	0 (0)
Arterial TE	7 (22.6)	0 (0)	0 (0)
Venous TE	22 (71)	0 (0)	0 (0)
Arterial and venous TE	2 (6.4)	0 (0)	0 (0)
*Pregnancy complications*^a^*, n (%)*	11 (35.5)	6 (37.5)	0 (0)
aPLAs, n (%)			
LA alone^b^	2 (6.5)	8 (50)	
LA+ anti–ß2GPI^c^	0 (0)	0 (0)	
LA+ aCL^c^	12 (38.7)	3 (18.8)	
LA+ anti-ß2GPI + aCL (triple positivity)^c^	17 (54.8)	5 (31.2)	
*Antithrombotic agent, n (%)*	24 (77.4)	4 (25)	0 (0)
VKA	19 (61.3)	1 (6.2)^d^	0 (0)
LMWH	2 (6.5)	0 (0)	0 (0)
DOACs	2 (6.5)	0 (0)	0 (0)
LDA	5 (16.1)	3 (18.8)^e^	0 (0)
Clopidogrel	1 (3.2)	0 (0)	0 (0)
None	7 (22.6)	12 (75)	0 (0)
*Concomitant ARD, n (%)*	8 (25.8)	3 (18.8)	0 (0)
*Hypertension, n (%)*	10 (32.3)	6 (37.5)	8 (17)
*Hyperlipidaemia, n (%)*	4 (12.9)	3 (18.8)	1 (2.1)
*Diabetes mellitus type 2, n (%)*	2 (6.5)	1 (6.3)	0 (0)
*Hydroxychloroquine, n (%)*	3 (9.7)	0 (0)	0 (0)

*y* years, *n* number, *IQR* interquartile range, *LA* lupus anticoagulant, *TE* thromboembolism, *ß2GPI* beta-2 glycoprotein, *aCL* anti-cardiolipin antibodies, *VKA* vitamin K antagonist, *LMWH* low-molecular-weight heparin, *DOACs* direct oral anticoagulants, *LDA* low-dose aspirin, *ARD* autoimmune rheumatic disease

^a^Patients with at least one pregnancy (LA+ TE+ n = 20; LA+ TE− *n* = 12; Healthy *n* = 22). Pregnancy complications were defined according to current criteria^1^

^b^LA alone defined as the absence of IgG/IgM anti-β2GPI and aCL

^c^Cutoff: anti-β2GPI >8 GPL/MPL U/mL, aCL >40 GPL/MPL U/mL

^d^Due to atrial fibrillation

^e^Due to cardiovascular risk

### Determination of lupus anticoagulant (LA)

Diagnosis of LA followed the SSC/ISTH recommendations^17,18^. For LA determination, venous blood was drawn into 3.5 ml sodium citrate (0.129 mM citrate) vacuum tubes (Vacuette, Greiner-Bio One, Kremsmünster, Austria) and centrifuged at 2500 × *g* for 15 min at 15 °C twice. A lupus-sensitive activated partial thromboplastin time (aPTT-LA) and a diluted Russell’s viper venom time were used as screening tests. If one or both screening tests were prolonged, samples were further analyzed and confirmatory tests were performed, as described in detail by Wenzel et al.^19^. When the confirmatory test at this visit was not definitely positive, LA was still regarded as positive when the Rosner index, calculated as 100× (clotting times of the 1:1 mixture - normal plasma)/patient’s plasma was higher than 15, as described by Rosner et al.^20^. Confirmatory assays used were the StaClot LA (Diagnostica Stago, Asniere sur Seine, France) and the diluted Russell’s viper venom time-LA Confirm (Life Diagnostics, Clarkston GA, USA).

### Determination of aCLs and anti-β2GPI antibodies

IgG and IgM antibodies against aCL and anti-β2GPI were determined with indirect solid-phase enzyme immunoassays. The Varelisa Cardiolipin test (Pharmacia, Uppsala, Sweden) was performed semi-automatically using a Tecan Genesis liquid-handling system (Tecan Group Ltd, Maennedorf, Switzerland) from 2001 to September 2005. From October 2005 until October 2006 the Orgentec Cardiolipin test and afterwards the Orgentec β2-GPI test (both Orgentec, Mainz, Germany) was used on a fully automated BEP2000 Advance System (Siemens Healthcare Diagnostics, Marburg, Germany). All assays were performed according to the manufacturers’ instructions. According to the guidelines, the results were reported to be positive if >40 GPL/MPL U/mL for the Varelisa Cardiolipin and the Orgentec Cardiolipin test and >8 GPL/MPL U/mL (corresponding to the 99^th^ percentile of healthy controls) for anti-β2GPI IgG and IgM.

### Blood sampling, platelet and plasma isolation

For platelet isolation, venous blood was drawn into 3.5 ml CTAD (0.129 mM trisodium citrate, 15 mM theophylline, 3.7 mM adenosine, 0.198 mM dipyridamole) tubes (Vacuette, Greiner-Bio One, Kremsmünster, Austria) to avoid post-sampling platelet activation. Whole blood was centrifuged at 120 × *g* for 20 min at room temperature (RT) with the centrifugation brake off to avoid contamination with other blood cells.

Afterward, platelet-rich plasma was transferred into a fresh tube containing prostacyclin I2 (0.8 µM) to avoid platelet aggregation and degranulation during the following washing process. Platelets were then pelleted by centrifugation (3000 × *g*, 3 min), washed twice in phosphate-buffered saline containing prostacyclin I_2_ (0.8 µM) and finally pelleted, shock-frozen in liquid nitrogen and stored at −80 °C. To obtain platelet-poor plasma, venous blood was drawn into 3.5 ml sodium citrate (0.129 mM citrate) vacuum tubes (Vacuette, Greiner-Bio One, Kremsmünster, Austria), centrifuged at 2500 × g for 15 min at 15 °C and stored at −80 °C. All platelet and plasma samples were further processed within 6 months to avoid stability problems.

### Sample preparation for 2D-DIGE analysis

The platelet pellet was resolubilized in urea-sample buffer (7 M urea, 2 M thiourea, 4% CHAPS, 20 mM Tris–HCl pH 8.8) and incubated for 2 h at 4 °C under agitation (800 rpm). Protein concentration was determined using a Coomassie brilliant blue protein assay kit (Pierce Biotechnology, Rockford, IL, USA). The internal standard (IS) for 2D-DIGE analysis was made by pooling the same amount of total protein from each study sample. Aliquots of IS and individual study samples were stored at −80 °C.

### Platelet proteome analysis by 2D-DIGE

Urea-sample buffer–resolubilized platelet proteins were labeled with fluorescent cyanine dyes (CyDyes®, GE Healthcare, Uppsala, Sweden) according to the manufacturer's instructions (except at a reduced labeling concentration: 5 pM of dye per μg of protein). The IS was always labeled with Cy2, while Cy3 and Cy5 were alternatingly used for samples. Isoelectric focusing, electrophoresis and gel image processing were performed as previously described^21^. Briefly, 24 cm pH 4–7 IPG-Drystrips (GE Healthcare) were rehydrated for 12–15 h with 450 μL rehydration buffer (7 M urea, 2 M thiourea, 70 mM DTT, 0.5% ampholyte pH 4–7) mixed with 12 μg of each Cy-labeled sample. The 2-dimensional separation was performed by sodium dodecyl sulfate–polyacrylamide gel electrophoresis on 11.5% gels (25.5 × 20.5 cm) using the following protocol: 35 V for 1 h, 50 V for 1.5 h and 110 V for 16.5 h at 10 °C.

### 2D-DIGE image analysis

For spot detection, gels were scanned at a resolution of 100 μm using a Typhoon 9410 imager (GE Healthcare, Uppsala, Sweden) with subsequent image analysis using the DeCyder software (version 7.2; GE Healthcare, Uppsala, Sweden). Only protein spots that could be manually matched with the IS in at least 80% of all gels and were significantly changed between our study cohorts (calculated by one-way ANOVA) were defined as altered spots.

### Protein identification

To identify proteins by mass spectrometry (MS), 250 μg unlabeled proteins were separated by 2D-DIGE and visualized by MS-compatible silver staining^22^. Altered protein spots were excised, destained, reduced, alkylated and tryptically digested. Liquid chromatography (LC)-MS analysis was performed as previously described^23^. In brief, peptides were separated using a Dionex Ultimate 3000 RSLC nano-HPLC system (Thermo Scientific) equipped with a preconcentration and desalting cartridge where 0.1% TFA was used as transport liquid, and proteins were separated on an Acclaim PepMap RSLC column (250 mm × 75 μm, C18, 2 μm, 100 Å; Thermo Scientific) by elution with a 59 min linear gradient from 4 to 45% of solvent B using a flow rate of 500 nL/min (solvent A: 0.1% TFA, solvent B: acetonitrile/ddH2O/formic acid, 80/20/0.1% (v/v)). A QqTOF mass spectrometer oTOF compact from Bruker Daltonics (Billerica, MA, United States) equipped with a nanoflow CaptiveSpray ionization device was used for in-line, bottom-up proteomics and controlled by the oTOF control software v3.4 (build16). To achieve high mass accuracy, an internal calibrant (hexakis-(1H,-1H,-4H-hexafluorobutyloxy)-phosphazine (Agilent Technologies)) was used during the runs. MS1 and MS/MS fragmentation was set to the following parameters: cycle time was 3 s, scan range for precursor recording was 50−2,200 *m/z*, spectrum rate was 2 Hz, dry gas was 3 L/min, and dry temperature was 150 °C. Peptide assignments were conducted using Proteinscape software v.3.1.5 474 (Build 0140711-1459, Bruker Daltonics) equipped with the Mascot algorithm version 2.5 (Matrixscience, MA), and peak lists were generated with Compass Data Analysis software v4.2 (Build 395, Bruker Daltonics). For protein searching, the following parameters were used: database: UniProtKB/Swiss-Prot; species: human; enzyme specificity: trypsin; peptide tolerance ± 10 ppm; MS/MS tolerance ± 0.05 Da; number of considered 13 C atoms: 1; charge states: 1 + –3 + ; up to two missed cleavages; fixed modifications: carbamidomethylation (C); variable modifications: deamidation (N, Q), oxidation (M), phosphorylation (S, T, Y), acetylation (K, N-term). The applied probability score of the search algorithm was set to *p* < 0.05.

### Citrullinated histone H3 ELISA

Citrullinated histone H3 (H3Cit) ELISA was performed as previously described^24,25^. In brief, 96-well plates were coated with an anti-histone antibody (Cell Death Detection ELISA; Sigma–Aldrich, St Louis, MO, USA) overnight at 4 °C. H3Cit standards (recombinant human peptidylarginine deiminase 4 (PAD4) (Cayman Chemicals, Ann Arbor, MI, USA) and recombinant human histone H3.1 (New England Biolabs, Evry, France)) and plasma samples were incubated at RT for 1.5 h and then washed with PBS containing 0.05% Tween-20. Next, anti-H3Cit antibody (1:1000 ab5103; Abcam, Cambridge, MA, USA) was added for 1.5 h. After another washing step, anti-rabbit horseradish peroxidase (HRP)-conjugated antibody (1:5000 goat anti-rabbit IgG HRP; Bio-Rad Laboratories, Hercules, CA, USA) was incubated for 1 h, washed again and incubated with 3,3’,5,5’ -tetramethylbenzidine (Sigma–Aldrich, St Louis, MO, USA) for 25 min. Finally, the reaction was stopped with 2% sulfuric acid, and absorbance at 450 nm was measured using a Multiskan Spectrum microplate reader (Thermo Scientific Inc., Bremen, Germany).

### Protein disulfide isomerase A1 (P4HB) ELISA

P4HB was analyzed from all patient and control plasma by ELISA according to the manufacturer’s instructions (Human PDI/P4HB ELISA Kit, LSBio LifeSpan BioSciences, Inc., Seattle, WA, USA).

### One- and two-dimensional western blot analysis

One- and two-dimensional western blot (WB) analysis was performed as previously described^4^. Platelets for 1D WB analysis were mixed with sample buffer (150 mM Tris-HCl pH 6.8, 7.5% SDS, 37.5% glycerol, bromine phenol blue, 125 mM DTT), boiled for 5 min at 95 °C and centrifuged at 20,000 × *g* for 3 min. Thereafter, 12 μg/lane of this protein sample was loaded and separated on an 11.5% SDS gel (20 × 10 cm; 50 V for 20 min and 100 V for 150 min) and subsequently blotted (75 V for 120 min) on a polyvinylidene difluoride membrane (FluoroTrans® W, Pall, East Hills, NY, USA). For protein quantification, a 1D WB ion-based ruthenium (Sigma–Aldrich St. Louis, MI USA) whole-protein stain was performed (dilution 1:100 000, overnight at 4 °C)^5^, followed by scanning with a Typhoon FLA 9500 imager (GE Healthcare, Uppsala, Sweden). Subsequently, membranes were blocked in 5% nonfat dry milk (BioRad, Hercules, CA, USA) in PBS containing 0.3% Tween-20 overnight at 4 °C. On the next day, membranes were washed and incubated with primary antibodies for 2 h at RT (monoclonal protein disulfide isomerase A1 (P4HB), clone RL90, 1:1000; monoclonal leukocyte elastase inhibitor (SERPINB1) clone EPR13305(B), 1:1000, both from Abcam, Cambridge, UK). After washing, membranes were incubated with DyLight 650–conjugated secondary antibody (1:500, Novus Biologicals, Littleton, CO, USA) for 1.5 h at RT in the dark and detected with a Typhoon FLA 9500 imager (GE Healthcare, Uppsala, Sweden). The antibody signals of P4HB and SERPINB1 were normalized by the ruthenium fluorescence signal from the 40 kDa to 100 kDa bands and quantified with ImageQuant 8.0 (GE Healthcare, Uppsala, Sweden).

For 2D WB analysis, 36 μg of resolubilized Cy2-labeled platelet proteins were separated by isoelectric focusing on either a 7 cm pH 3–10 or a 24 cm pH 4–7 IPG strip (GE Healthcare, Uppsala, Sweden) in the first dimension and according to the molecular weight by 11.5% SDS-PAGE in the second dimension. Complete 8.6 × 6.8 cm SDS gel and relevant isoelectric point regions from the 24 cm SDS gel were blotted (75 V for 120 min) on a nitrocellulose membrane (Pall, East Hills, NY). Subsequently, membranes were blocked, incubated with primary (P4HB and SERPINB1) and secondary antibodies and detected as described for 1D WB analysis.

### Biological pathway analysis

The protein-protein interaction (PPI) networks were drawn and their functional enrichment evaluated in Cytoscape software version 3.6.1^26^. Only those proteins with an adjusted *p*-value ≤ 0.05 in each comparison (ANOVA) were considered. The data source for the PPI networks was the protein query of the STRING database^27^ (accessed on 24.07.2018), with default settings (score = 0.4; maximal additional interactors = 0). For the functional enrichment (Gene Ontology Biological Processes; default settings; accessed on 24.07.2018) of the PPI networks, the STRING Enrichment app^27^ was used. Each biological process is represented by a specific color. The enrichment graphs were made in ggplot2^28^.

### Statistics

For statistical analysis, only protein spots that could be manually matched with the IS in at least 80% of all gels were included, which resulted in the statistical analysis of 258 protein spots. From these 258 protein spots, 44 proteins were significantly altered between the study cohorts, calculated by multiple comparisons–corrected one-way analysis of variance (one-way ANOVA) for more than two groups and the unpaired *t*-test for post-hoc comparison. Standardized abundance of protein spots, identified in more than one spot and regulated in the same direction, were summed up, since general pan-antibodies against particular proteins do not differentiate between altered protein species. For normalization, this generated sum was divided by the volume of the summarized IS. Within this project, we validated the antibody specificity of protein disulfide isomerase A1 (P4HB) by 2D WB and found that the antibody detected all altered isoforms (Supplementary Fig. 2a, Supplementary Table 1). The statistics of the summed proteins (31 protein spots) are outlined in Table 2, and the statistics of all 45 significantly altered protein spots are listed in Supplementary Table 1. However, these generated spot sums may not generally reflect the overall volume/concentration of the respective proteins (there may still be other spots of these proteins in the gel, either unchanged in abundance or filtered out in statistical evaluation). This limitation should be mentioned because this approach may create discrepancies between 1D WB or ELISA and 2D-DIGE results.

**Table 2:** Altered platelet proteins between LA+ TE+ patients, LA+ TE− patients and healthy controls analyzed by 2D-DIGE.

Protein name	Gene name	LA+ TE+ /healthy		LA+ TE+ /LA+ TE−		LA+ TE−/healthy	
		Average fold-change	p-value [adjusted]	Average fold-change	p-value [adjusted]	Average fold-change	p-value [adjusted]
60 kDa heat shock protein	HSPD1	1.05	0.1045	**1.14**	**0.0042**	0.93	0.0825
78 kDa glucose-regulated protein^a^	HSPA5	**1.11**	**0.0047**	**1.19**	**0.0002**	0.93	0.0528
Actin	ACTB	1.18	0.1873	**0.51**	**0.0276**	**2.31**	**0.0074**
Albumin^a^	ALB	0.92	0.1138	**0.76**	**0.0013**	**1.21**	**0.0271**
Apolipoprotein A-I	APOA1	**0.86**	**0.0097**	0.90	0.1716	0.96	0.5661
ATP synthase subunit beta	ATP5B	1.09	0.6948	**1.11**	**0.0321**	0.98	0.0662
Bridging integrator 2	BIN2	0.86	0.3233	1.29	0.0671	**0.67**	**0.0232**
Calreticulin^a^	CALR	**1.10**	**0.0019**	**1.16**	**0.0033**	0.95	0.6012
Chloride intracellular channel protein 1	CLIC1	0.60	0.2305	**0.23**	**0.0033**	2.67	**0.0297**
Fermitin family homolog 3	FERMT3	**0.90**	**0.0180**	1.07	0.4783	0.76	**0.0261**
Fibrinogen beta chain	FGB	**1.21**	**0.0097**	1.01	0.8706	1.19	**0.0297**
		**1.09**	**0.0255**	**1.13**	**0.0177**	0.97	0.5661
		**1.31**	**0.0112**	1.29	0.1716	1.02	0.5661
		**1.27**	**0.0130**	1.30	0.1716	0.98	0.6012
Heat shock protein HSP 90-alpha	HSP90AA1	**1.10**	**0.0026**	1.06	0.1493	1.04	0.3496
Integrin alpha-6^a^	ITGA6	**0.89**	**0.0097**	**0.85**	0.0018	1.06	0.2110
Leukocyte elastase inhibitor	SERPINB1	**0.90**	**0.0164**	0.94	0.1754	0.97	0.6012
Microtubule-associated protein RP/EB family member 2^a^	MAPRE2	1.13	0.1249	0.87	0.2678	**1.31**	**0.0297**
Myosin 9^a^	MYH9	0.80	0.0676	**0.63**	**0.0013**	**1.29**	**0.0298**
Myosin regulatory light polypeptide 9	MYL9	1.09	0.2043	**1.23**	**0.0248**	0.88	0.2110
Nucleosome assembly protein 1-like 1	NAP1L1	1.02	0.5365	**1.23**	**0.0033**	**0.83**	**0.0232**
Proteasome activator complex subunit 1	PSME1	**1.17**	0.0014	**1.15**	**0.0161**	1.02	0.7921
Protein disulfide isomerase A1^a^	P4HB	**1.10**	0.0047	**1.16**	**0.0016**	0.95	0.2835
Protein disulfide isomerase A6	PDIA6	**1.07**	0.0047	**1.13**	**0.0043**	1.00	0.5661
Ras-related protein Rab-27B	RAB27B	0.91	0.1723	1.12	0.1716	**0.81**	**0.0280**
Translationally controlled tumor protein	TPT1	1.08	0.1362	1.17	0.0177	0.93	0.2110
Tropomyosin alpha-3 chain	TPM3	0.95	0.6979	1.28	0.0160	**0.74**	**0.0212**
Vinculin	VCL	**1.37**	**0.0115**	1.45	0.0800	0.94	0.9631
		**1.26**	**0.0097**	1.23	0.1716	1.03	0.6012
		**1.25**	**0.0014**	**1.32**	**0.0106**	0.95	0.9144
		**0.75**	**0.0385**	1.03	0.7263	0.73	0.0585

Differentially regulated proteins were considered significant when the fold-change differed by at least 10% and the p-value was ≤ 0.05. A fold-change above 1 indicates that the protein is upregulated, whereas a fold-change below 1 indicates that the protein is downregulated in the first stated group. For detailed protein description, refer to Supplementary Table 1

Statistically significant correlations are highlighted in bold

*LA* lupus anticoagulant, *TE* thromboembolism

^a^Proteins identified in more than one spot and regulated in the same direction. The standardized abundances of the 2D-DIGE spots were summed and divided by the volume of the summarized IS for normalization. Fibrinogen beta chain and vinculin were not regulated in the same direction; therefore, all isoforms are stated. All proteoforms are listed in Supplementary Table 1

All statistical calculations for 2D-DIGE analysis were false discovery rate (FDR)-corrected by the Benjamini-Hochberg procedure^29^ for multiple comparisons. One-way ANOVA was FDR-corrected for 258 protein spots using the EDA module of the DeCyder software (version 7.2, GE Healthcare, Uppsala, Sweden); the unpaired post-hoc *t*-test was corrected for 31 proteins using an FDR online calculator (Seed-based d Mapping, https://www.sdmproject.com/utilities/?show=FDR). Differences in protein abundance were considered significant when the fold-change was ≥1.1 or ≤0.9 and the corrected p-value was ≤0.05.

Other laboratory parameters are described by the median and the interquartile range (IQR), indicating the 25th–75th percentile, for each study group. Here, differences between groups were determined by the Kruskal-Wallis test, and the Wilcoxon-Mann-Whitney U-Test was used as a post-hoc test. The relation between aPLA levels and protein abundances was exploratorily assessed by Spearman’s rank correlation coefficient, as aPLA levels did not show linearity. Additional statistical calculations were made with SPSS version 17.0.2 (SPSS Inc., Chicago, USA). Graphs were made with GraphPad Prism 6 (GraphPad Software, Inc. San Diego California, USA).

## Results

### Patient characteristics

To investigate whether the platelet proteome is affected in patients with persistent LA, 47 LA-positive patients were studied. At the time of inclusion, 31 patients had a history of TE (LA+ TE+) and 16 were LA-positive without prior thrombotic manifestations (LA+ TE−). One patient within the LA+ TE+ group suffered from CAPS with an acute thrombotic event at the time of blood sampling. Within the LA+ TE+ group, 22 patients had a history of venous thromboembolism (VTE), 7 had arterial thromboembolism (ATE) and 2 had both VTE and ATE. Twenty-eight patients received antithrombotic agents, of whom 20 had vitamin K antagonists, two patients had low-molecular-weight heparin, two patients received a direct oral anticoagulant, and eight patients received antiplatelet therapy including low-dose aspirin and clopidogrel, whereas 19 patients received no anticoagulant therapy. We did not detect an influence of antithrombotic agents on platelet protein abundance (Supplementary Table 2). Detailed patient characteristics are shown in Table 1.

### Differential proteomic analysis of LA-positive patients with or without a history of thrombosis and healthy controls

**Fig. 1:**
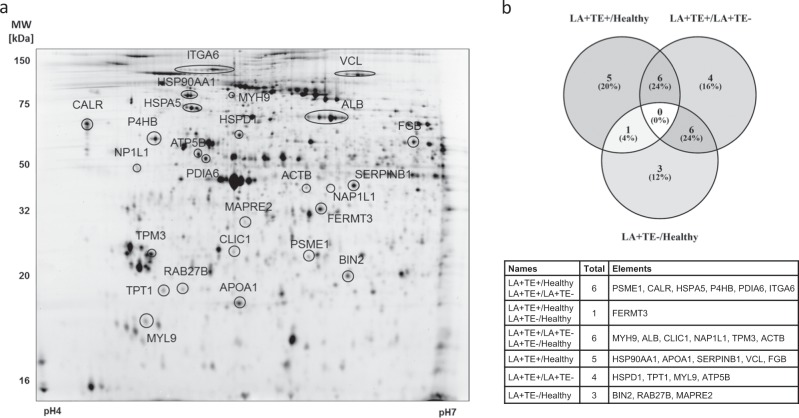
Altered platelet proteins analyzed by 2D-DIGE. **a** Representative 2D-DIGE gel with altered platelet proteins between LA+ TE+ patients compared to control groups. Detailed descriptions of the highlighted proteins are listed in Table 1. **b** Venn diagram^58^ illustrating the overlap between the altered protein spots between LA+ TE+ /Healthy, LA+ TE+ /LA+ TE− and LA+ TE−/Healthy. MW molecular weight, kDa kilodalton, 2D-DIGE two-dimensional differential in-gel electrophoresis, LA lupus anticoagulant, TE thromboembolism.

To identify thrombosis-related alterations in platelets from LA-positive patients, we designed a 2D-DIGE-based platelet proteome analysis in which LA-positive patients with or without a history of TE and healthy controls were compared. On average, 3088 spot events were detected per gel, and after manually matching of only well-defined protein spots matched in at least 80% of all gels, 258 proteins were statistically evaluated. Of these proteins, 44 were significantly altered between the study groups (one-way ANOVA, FDR-corrected, *p* ≤ 0.05, Fig. 1a, Supplementary Table 1). Nine proteins were present in more than one spot, and those that were regulated in the same direction were summarized, leading to 25 different proteins (or 31 final protein spots) (Table 2, summarized proteins are highlighted). From these LA-related proteome changes, 12 proteins were altered between LA+ TE+ and healthy controls, 16 proteins between LA+ TE+ and LA+ TE− and 10 proteins between LA+ TE− and healthy controls (Fig. 1b).

In a subsequent analysis, we investigated differences in the protein abundance between LA-positive patients with a history of ATE and LA-positive patients with a history of VTE and found that 5 proteins were significantly altered between those groups (Supplementary Table 3).

Among the significantly altered protein spots, we selected protein disulfide isomerase A1 (P4HB) and leukocyte elastase inhibitor (SERPINB1) for validation of LA-related abundance changes (Supplementary Figs. 2a–c and 3a–c). These proteins were selected because they were only altered in LA+ TE+ patients and have already been described to play a role in thrombus development from two completely different mechanistic perspectives^30–32^.

### STRING pathway analysis

Unbiased STRING pathway analysis was used to identify the possible involvement of altered proteins in biological pathways and processes. STRING pathway analysis computed that platelet activation and blood coagulation pathways were altered in LA-positive patients with or without a history of TE, whereas platelet degranulation was only altered in LA+ TE+ patients. Furthermore, STRING pathway analysis comparing LA-positive patients with TE, LA-positive patients without TE and healthy controls indicated an involvement of altered proteins in the regulation of biological quality and protein folding, representing a cellular stress response (Supplementary Fig. 1a–c).

### Association of altered platelet proteins with aPLAs in patients positive for LA

Associations of altered platelet proteins with aPLAs showed several weak to even moderate statistically significant correlations, predominately of proteins involved in pathways of the unfolded protein response, endoplasmic reticulum stress and antigen processing/presentation (P4HB, PDIA6, CALR, HSPA5, and HSP90AA1) (Table 3).

**Table 3:** Spearman correlation coefficients of altered platelet proteins and plasma levels of H3Cit and P4HB with aPLAs.

Protein name	Gene name	beta2IgG		beta2IgM		ACAIgG		ACAIgM		aPTT-LA	
		rho	p-value	rho	p-value	rho	p-value	rho	p-value	rho	p-value
60 kDa heat shock protein	HSPD1	0.17	0.10189	0.15	0.14360	0.17	0.09754	0.15	0.14584	0.04	0.68816
78 kDa glucose-regulated protein	HSPA5	**0.32**	**0.00197**	0.17	0.10405	**0.25**	**0.01634**	**0.26**	**0.01254**	0.13	0.20740
Actin	ACTB	0.19	0.08615	0.00	0.99256	**0.24**	**0.02999**	0.05	0.62676	**0.237**	**0.03334**
Albumin	ALB	−0.11	0.30463	0.18	0.08227	−0.03	0.78719	0.13	0.21880	0.00	0.97263
Apolipoprotein A1	APOA1	**−0.26**	**0.01510**	−0.07	0.54604	−**0.32**	**0.00291**	−0.14	0.19751	−**0.25**	**0.02460**
ATP synthase subunit beta	ATB5B	0.10	0.34481	0.10	0.34685	0.07	0.50187	0.10	0.33366	−0.03	0.75926
Bridging integrator 2	BIN2	−0.09	0.41266	−**0.23**	**0.02367**	−0.13	0.22210	−**0.23**	**0.02925**	−**0.28**	**0.00674**
Calreticulin	CALR	**0.39**	**0.00013**	**0.22**	**0.03227**	**0.33**	**0.00122**	**0.27**	**0.01003**	**0.22**	**0.03549**
Chloride intracellular channel protein 1	CLIC1	−0.16	0.13441	0.02	0.83857	−0.05	0.64154	−0.03	0.79319	−0.01	0.91898
Fermitin family homolog 3	FERMT3	−0.19	0.06772	−0.12	0.26248	−**0.21**	**0.04817**	−0.14	0.19047	−**0.33**	**0.00155**
Fibrinogen beta chain	FGB	**0.24**	**0.02130**	0.17	0.10211	**0.27**	**0.01106**	**0.22**	**0.04155**	**0.35**	**0.00103**
		0.16	0.15117	0.12	0.25037	0.12	0.25475	0.14	0.20021	0.16	0.13852
		0.14	0.19122	0.05	0.61319	0.14	0.16865	0.07	0.53253	0.17	0.09965
		**0.29**	**0.00653**	**0.24**	**0.02868**	**0.24**	**0.02427**	**0.25**	**0.02115**	0.09	0.43085
Heat shock protein HSP 90-alpha	HSP90AA1	**0.29**	**0.00520**	0.20	0.05763	**0.38**	**0.00018**	**0.26**	**0.01184**	**0.28**	**0.00707**
Integrin alpha-6	ITGA6	−**0.21**	**0.04766**	−0.09	0.37817	−0.14	0.19038	−0.15	0.15803	−0.08	0.47165
Leukocyte elastase inhibitor	SERPINB1	−0.20	0.05664	−0.04	0.71912	−0.16	0.11436	−0.07	0.47704	−0.16	0.13450
Microtubule-associated protein RP/EB family member 2	MAPRE2	0.15	0.16008	**0.22**	**0.04858**	0.12	0.26731	0.19	0.08717	**0.24**	**0.02602**
Myosin 9	MYH9	−0.14	0.16585	−0.10	0.33328	−0.11	0.27362	−0.17	0.09670	−0.01	0.93748
Nucleosome assembly protein 1-like 1	NAP1L1	0.06	0.59095	0.04	0.70443	0.01	0.95578	0.06	0.58759	−0.01	0.90886
Proteasome activator complex subunit 1	PSME1	**0.26**	**0.01136**	**0.24**	**0.01895**	**0.23**	**0.02776**	**0.24**	**0.01957**	**0.27**	**0.00963**
Protein disulfide isomerase A1	P4HB	**0.32**	**0.00201**	**0.22**	**0.03279**	**0.24**	**0.02017**	**0.26**	**0.01355**	0.17	0.11490
Protein disulfide isomerase A6	PDIA6	**0.26**	**0.01082**	0.15	0.16264	**0.23**	**0.02398**	0.19	0.06515	0.16	0.12280
Ras-related protein Rab-27B	RAB27B	−0.10	0.36735	−0.11	0.29290	−0.12	0.24516	−0.12	0.25889	−0.27	**0.00951**
Translationally controlled tumor protein	TPT1	0.13	0.21697	0.12	0.24945	0.13	0.22414	0.15	0.14543	0.07	0.50463
Tropomyosin alpha-3 chain	TPM3	0.02	0.89045	−0.09	0.42401	−0.04	0.73541	−0.09	0.42459	−**0.23**	**0.03298**
Vinculin	VCL	**0.31**	**0.00635**	0.17	0.14116	**0.27**	**0.01620**	**0.26**	**0.02380**	0.20	0.08571
		**0.32**	**0.00191**	0.20	0.05917	**0.32**	**0.00234**	**0.27**	**0.00868**	**0.29**	**0.00667**
		**0.28**	**0.01027**	0.18	0.10479	**0.27**	**0.01329**	**0.25**	**0.02180**	**0.31**	**0.00420**
		−0.17	0.10760	−**0.25**	**0.01770**	−0.19	0.07469	**−0.25**	**0.01675**	**−0.33**	**0.00145**
H3Cit ELISA [ng/ml]		**0.21**	**0.04758**	0.18	0.07782	0.18	0.08715	**0.22**	**0.03193**	**0.29**	**0.00506**
P4HB ELISA [ng/ml]		**0.41**	**0.00004**	**0.32**	**0.00164**	**0.41**	**0.00005**	**0.35**	**0.00064**	**0.38**	**0.00024**

Statistically significant correlations are highlighted in bold

*aPLAs* antiphospholipid autoantibodies

### Protein disulfide isomerases (PDIs) are elevated in platelets and plasma of LA+ TE+ patients compared to control groups

**Fig. 2:**
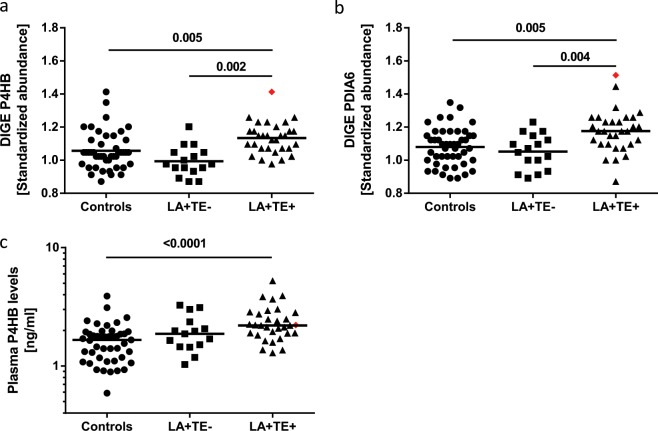
Analysis of protein disulfide isomerases (P4HB and PDIA6) in human platelets and plasma between LA+ TE+ patients and control groups. **a** LA+ TE+ patients had significantly increased protein abundance of P4HB in platelets compared to healthy controls (*p* = 0.005) or patients without a history of TE (*p* = 0.002). **b** Standardized abundance of PDIA6 was significantly increased in LA+ TE+ patients compared to healthy controls (*p* = 0.005) and LA+ TE− patients (*p* = 0.004). **c** In addition, P4HB levels in plasma were significantly elevated in LA+ TE+ patients compared to healthy controls (*p* = ≤ 0.0001). Each dot represents a measurement of a single patient. The patient with CAPS is visualized as a red rhombus. The line between the dots of Fig. 2a, b represents the mean (FDR-corrected *t*-test) and of Fig. 2c the median (Wilcoxon-Mann-Whitney-U-Test). LA lupus anticoagulant, TE thromboembolism, 2D-DIGE two-dimensional differential in-gel electrophoresis, CAPS catastrophic antiphospholipid antibody syndrome.

PDIs are involved in thrombus formation in vivo^30^. Platelet proteome analysis revealed that two members of the PDI family were significantly altered between our study groups (Table 2). LA+ TE+ patients showed significantly increased protein abundances compared to healthy controls (P4HB: *p* = 0.005; PDIA6: *p* = 0.005) or LA+ TE− patients (P4HB: *p* = 0.002; PDIA6: *p* = 0.004*)* (Fig. 2a, b, Table 2). P4HB abundance changes detected with 2D-DIGE were validated by qualitative 2D and quantitative 1D WB analysis (Supplementary Fig. 2b, c). Since activated platelets release PDIs^33^, we additionally analyzed the plasma P4HB levels by ELISA. LA+ TE+ patients had significantly elevated plasma P4HB levels (2.20 ng/ml, IQR: 1.89–2.82) compared to healthy controls (1.68 ng/ml, IQR: 1.17–1.94, *p* = ≤ 0.0001) (Fig. 2c). No significant difference could be detected between LA+ TE+ and LA+ TE− patients (1.92 ng/ml, IQR: 1.48–2.68, *p* = 0.19) or between LA+ TE− patients and healthy controls (*p* = *0.06*). The highest abundances of platelet P4HB and PDIA6 (red rhombus shown in Fig. 2a, b) were measured in the patient with CAPS. P4HB plasma levels (red rhombus in Fig. 2c) were also but moderately increased in the patient with CAPS compared to the control groups. Consistently positive correlations were observed between platelet/plasma P4HB and several APLAs, as shown in Table 3.

### Concentrations of leukocyte elastase inhibitor (SERPINB1) are decreased in platelets, and neutrophil extracellular traps (NETs) are increased in the plasma of LA+ TE+ patients compared to control groups

**Fig. 3:**
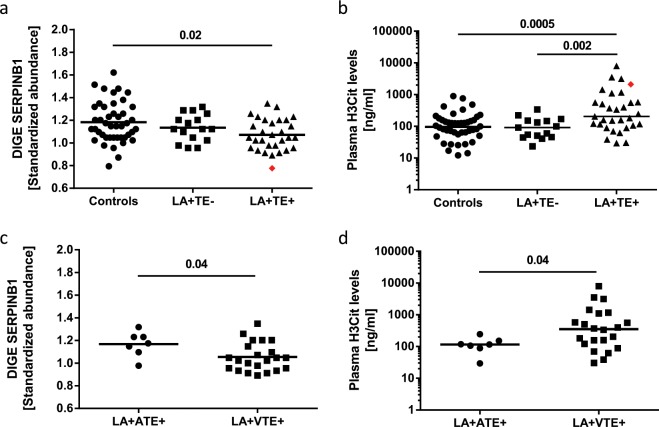
Analysis of leukocyte elastase inhibitor (SERPINB1) levels in platelets and neutrophil extracellular traps in the plasma of LA-positive patients with or without a history of TE and healthy controls. **a** Standardized abundance of SERPINB1 in the human platelet proteome showed that LA+ TE+ patients had significantly lower protein abundance of SERPINB1 compared to healthy controls (*p* = 0.02) and nonsignificantly lower compared to patients without thrombosis (*p* = 0.18). **b** LA+ TE+ patients had significantly higher H3Cit levels [ng/ml] compared to healthy controls (*p* = 0.0005) or LA+ TE− patients (*p* = 0.002). **c** LA-positive patients with a history of VTE had a lower SERPINB1 abundance than LA-positive patients with a history of ATE (*p* = 0.04). **d** H3Cit plasma levels were significantly increased in LA-positive patients with a history of VTE compared to LA-positive patients with a history of ATE (*p* = 0.04). Each dot represents a measurement of a single patient. The patient with CAPS is visualized as a red rhombus. The line between the dots of Fig. 3a, c represents the mean (FDR-corrected *t*-test and unadjusted Student’s *t*-test) and of Fig. 3b, d the median (Wilcoxon-Mann-Whitney-U-Test). LA lupus anticoagulant, TE thromboembolism, 2D-DIGE two-dimensional differential in-gel electrophoresis, CAPS catastrophic antiphospholipid antibody syndrome.

SERPINB1 belongs to the protein superfamily of serine protease inhibitors. Its main function is the inhibition of neutrophil elastase, an important effector for NET formation (NETosis)^31^. LA+ TE+ patients had a lower protein abundance of SERPINB1 compared to healthy controls (*p* = 0.02) and a small, statistically nonsignificant reduction compared to LA+ TE− patients (*p* = 0.18) (Fig. 3a, Table 2). SERPINB1 abundance changes detected with 2D-DIGE were validated by qualitative 2D and quantitative 1D WB analysis (Supplementary Fig. 3b, c). To examine a possible functional outcome of the decreased SERPINB1 levels, we analyzed H3Cit, a NET-specific marker, from patient and control plasma. LA+ TE+ patients had significantly higher H3Cit levels (204.90 ng/ml, IQR: 106.46–575.12) compared to healthy controls (95.39 ng/ml, IQR 50.31–157.18; *p* = 0.0005) or LA+ TE− patients (75.80 ng/ml, IQR: 45.26–149.14, *p* = 0.002). No differences in H3Cit levels were detected between LA+ TE− patients and healthy controls (*p* = 0.59) (Fig. 3b). The patient with CAPS had the lowest SERPINB1 abundance in platelets (red rhombus shown in Fig. 3a) and a high H3Cit level in plasma (red rhombus shown in Fig. 3b). In a more detailed analysis, we detected that LA-positive patients with a history of VTE had a lower SERPINB1 abundance than LA-positive patients with a history of ATE (*p* = 0.04) (Fig. 3b, Supplementary Table 3). Likewise, we detected significantly increased H3Cit levels in LA-positive patients with a history of VTE (354.65 ng/ml, IQR: 122.07–1107.31) compared to LA-positive patients with a history of ATE (116.48 ng/ml, IQR: 88.74–152.01, *p* = 0.04) (Fig. 3d). Correlation coefficients were negative when platelet SERPINB1 levels were correlated with plasma aPLA levels; however, the associations were weak and statistically not significant. In contrast, plasma H3Cit levels did, albeit weakly, correlate with several APLAs, as shown in Table 3.

### Other thrombosis-related changes in platelets of LA+ TE+ patients compared to control groups

**Fig. 4:**
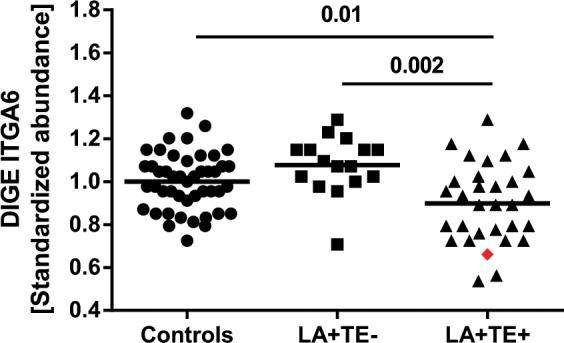
Standardized abundance of integrin α6 (ITGA6) in the human platelet proteome. Protein abundance of ITGA6 was significantly reduced in LA+ TE+ patients compared to healthy controls (*p* = 0.01) and patients without thrombosis (*p* = 0.002). Each dot represents a measurement of a single patient. The patient with CAPS is visualized as a red rhombus. The line between the dots represents the mean. FDR-corrected *t*-test was used to compare differences between groups. LA lupus anticoagulant, TE thromboembolism, 2D-DIGE two-dimensional differential in-gel electrophoresis, CAPS catastrophic antiphospholipid antibody syndrome.

Integrins are transmembrane receptors involved in cellular interactions. Platelets express several kinds of integrins, including integrin α6 (ITGA6), a platelet adhesion receptor for laminin^34^. We found that ITGA6 was significantly reduced in LA+ TE+ patients compared to healthy controls (*p* = 0.01) and to LA+ TE− patients (*p* = 0.002) (Fig. 4, Table 2). Additionally, the patient with CAPS showed strong abundance changes in other LA+ TE+ related platelet proteins (ALB, ATP5B, CALR, ITGA6, HSP90AA1, HSPA5, HSPD1, and PSME1; for full protein names, please refer to Table 2) compared to control groups.

## Discussion

Elevated platelet reactivity may contribute to increased thrombotic risk in patients with persistent positive LA. By investigating the proteome of platelets in LA-positive patients by 2D-DIGE, we aimed to discover their possible molecular and functional connection with thrombotic events. The comparative platelet proteome profiling of LA-positive patients with and without a history of thrombosis, matched to healthy controls, identified multiple altered proteins, expanding our current understanding of thrombus development in LA-positive patients.

STRING pathway analysis revealed that several LA-related platelet proteins were involved in platelet activation, degranulation and blood coagulation, which further strengthens the hypothesis that platelets from LA-positive patients are involved in the pathogenesis of thrombosis. Interestingly, platelet activation and blood coagulation pathways were differentially affected in patients with and without a history of thrombosis compared to healthy controls. Previous studies have also shown that platelets from LA-positive patients without a history of thrombosis were similar^35^ or even more activated compared to LA-positive patients with TE^36^. However, several platelet proteins already known to play a role in the increased risk of thrombosis were significantly altered only in LA-positive patients with a history of TE. These observations are emphasized by the extreme protein abundance changes of these platelet proteins in the patient with CAPS, such as the highest protein abundances of PDIs.

In general, family members of PDI, P4HB, PDIA6, and PDIA3 (data on PDIA3 not shown, increase of 8%, unadjusted *p* = 0.001; it did not meet our stringent statistical selection criteria) were significantly increased in the platelet proteome as well as in plasma (P4HB) of LA-positive patients with a history of TE. PDIs belong to the thiol isomerase family and are found in platelets, leukocytes and endothelial cells^37–39^, which release PDIs into the extracellular surroundings upon stimulation^40,41^. It is involved in platelet-mediated thrombus formation, aggregation and fibrin formation^30^ and is also elevated in platelets of patients with thalassemia, who have a high thrombotic risk^42^. In addition to its function in platelet activation, PDIs have been shown to cleave disulfide bonds in β_2_GPI, an autoantigen in APS^43^. These bonds appear in different redox states; the reduced form shows a protective function for endothelial cells, and the oxidized form is associated with increased thrombus formation^30^. APS patients have higher blood levels of oxidized β_2_GPI^44^, indicating the involvement of PDIs in β_2_GPI-related thrombus formation in APS patients. Moreover, it has been reported that APS patients have a significantly higher platelet-dependent baseline thrombin generation than healthy controls. Oral administration of isoquercetin inhibits PDI activity in plasma and diminishes platelet-dependent thrombin generation in healthy volunteers and patients with APS^45^. All these observations support a causal association of PDIs in the prothrombotic state of LA-positive patients and suggest that PDIs may become a specific treatment target in LA-positive patients.

Our findings that SERPINB1 protein levels are significantly reduced in platelets of LA-positive patients with a history of TE may be functionally related to increased formation of NETs and increased thrombotic risk^32^. SERPINB1 is a serine protease mainly described in the cytoplasm and granules of neutrophils but has also been detected in the extracellular space^46^, in platelets^47^ and in the platelet secretome^48^. SERPINB1 is one of the most efficient inhibitors and regulators of neutrophil elastase^49^, an important enzyme involved in NET formation. NETosis is an antimicrobial strategy that results in the externalization of decondensed chromatin containing granular and nuclear peptides^50^. NETs have also been identified as an important mediator of thrombosis^32^. SERPINB1 restricts NET generation in vitro and in vivo^31,51^. Accordingly, reduced SERPINB1 levels might be followed by increased NET formation. Our findings strengthen this hypothesis, as we found that LA-positive patients with TE had increased H3Cit plasma levels, indicating increased NET formation, which is in accordance with data from other groups^52,53^. Additionally, we detected that LA-positive patients with a history of VTE had significantly decreased platelet abundance of SERPINB1 and significantly increased H3Cit plasma levels compared to LA-positive patients with a history of ATE. This is in accordance with previous studies from our group, which showed that H3Cit levels are associated with the occurrence of VTE^24^ but is not associated with the risk of ATE in patients with cancer^54^, indicating that NETosis might be involved in the pathophysiology of venous but not arterial TE.

A significant reduction in the ITGA6 protein level was observed in platelets of LA-positive patients with TE. Platelet ITGA6, as part of the membrane integrin α6β1 complex, plays a promoting role in platelet adhesion/activation and ATE^55^. Accordingly, decreased integrin levels in platelets of LA-positive patients would be functionally contradictory for increased thrombotic risk. Nevertheless, in one of our previous studies, we compared the proteome of platelets and their released EVs upon activation. Interestingly, EVs contained higher levels of integrins compared to resting platelets^48^. Additionally, it was shown that APS patients have elevated plasma levels of EVs^56^. This evidence suggests that platelets in LA-positive patients are continuously activated and steadily shed EVs, which may finally lead to reduced platelet ITGA6 abundance.

In contrast to membrane ITGA6, proteins such as PDIs and SERPINB1 need to be translocated to the cell membrane or secreted from platelets to have an effect on thrombus development. In a previous study using shotgun proteomics, we detected PDIs and SERPINB1 in the secretome of TRAP-activated platelets^48^, indicating that platelets release those proteins upon activation.

To search for further functional associations with pathological hallmarks of APS, the newly characterized LA-related platelet proteins and plasma markers were correlated with aPLA levels. Several proteins showing a positive correlation with aPLAs act as chaperones in the endoplasmic reticulum, including PDI family members, GRP78 and CALR. These findings indicate the induction of the unfolded protein response, a cellular stress response related to endoplasmic reticulum stress^57^, and therefore higher cellular stress in LA-positive patients, which is in accordance with another study^48^. Interestingly, autoimmune diseases are associated with the unfolded protein response, which has been discussed as a potential pharmacological target to counteract autoantigen generation and presentation^57^.

Several limitations of the present study must be considered. There are limitations regarding the proteomic approach. The 2D-DIGE pH range 4-7 does not cover the whole pH range from 3 to 10, and low-abundance, very hydrophobic, very basic/acidic, and high-molecular-weight proteins are underrepresented. However, 2D-DIGE provides highly reliable quantitative results on differential protein expression. The selection of a narrower pH range (4–7) in a separating distance of 24 cm enables a better resolution of proteins and their pI-dependent posttranslational modifications. Moreover, 2D-DIGE technology with the inclusion of an IS has quite a low technical variation, with a CV of 7%^15^. Therefore, we chose 2D-DIGE technology for this quantitative clinical proteomics study in platelets of LA-positive patients. Nevertheless, a combination with other proteomic approaches, such as shotgun proteomics, could potentially reveal more and synergistic information on this important topic and should be considered for future studies.

Further shortcomings are the relatively small number of patients and the fact that some patients suffered from other diseases, such as autoimmune rheumatic disease (ARD), hypertension and diabetes mellitus, type 2. APS is a rare condition, and we are not able to distinguish the impact of comorbidities in our analysis due to the low number of patients. The inclusion of patients positive for LA only, but not of those positive for aCL or anti-ß2GPI antibodies only, could also be interpreted as a limitation. However, LA positivity is the highest risk factor for TE, and the diagnosis of the presence of LA follows strict guidelines. Furthermore, some patients received antithrombotic treatment. Hence, we tested the impact of antithrombotic agents as a confounding factor and could not find a significant impact.

To conclude, the results from this platelet proteomics study reveal specific alterations in the protein profiles of LA-positive patients, especially those with a history of TE, many of which are known to be associated with thrombus formation. Our findings further strengthen the hypothesis that platelets are involved in the thrombotic risk of LA-positive patients and could aid in developing effective treatment alternatives in addition to or instead of anticoagulation.

## Supplementary information

Supplemental data
