# Supplementary material for: Altered platelet proteome in lupus anticoagulant (LA)-positive patients—protein disulfide isomerase and NETosis as new players in LA-related thrombosis
Source: Exp Mol Med. 2020 Jan 20;52(1):66–78. doi: 10.1038/s12276-019-0358-4 (PMC7000701; doi:10.1038/s12276-019-0358-4)
Supplement: Supplementary file 1 — Supplemental data [file 12276_2019_358_MOESM1_ESM.pdf]

Altered platelet proteome in lupus anticoagulant (LA)-positive patients – Protein disulfide-isomerase and NETosis as new players in LA-related thrombosis

Authors:

Lena Hell[1], Kristina Lurger[1], Lisa-Marie Mauracher[1], Ella Grilz[1] Christina Maria Reumiller[2], Georg Johannes Schmidt[3], Huriye Ercan[1], Silvia Koder[1], Alice Assinger[2], José Basilio[2], Johanna Gebhart[1], Cihan Ay[1], Ingrid Pabinger[1*] and Maria Zellner[2*]

Supplementary Information

Within the Supplementary Information the authors provide more detailed data shown in tables and figures. Tables provide more detailed information on altered platelet proteins and additional statistical evaluations. Figures show STRING pathway analysis of altered platelet proteins as well as confirmatory Western Blot analysis of P4HB and SERPINB1.

Supplementary Tables:

Supplementary Table 1: Altered platelet proteins between LA+TE+ patients, LA+TE- patients and healthy controls analyzed by 2D-DIGE. Differentially regulated proteins were considered significant when fold change differed for at least 10% and the p-value was ≤0.05. A fold change above 1 indicates that the protein is increased, whereas a fold change below 1 indicates that the protein is decreased in the first stated group.

						LA+TE+/Healthy			LA+TE+/LA+TE-			LA+TE-/Healthy		
**Protein name**	**Gene name**	**Uniprot Number.**	**Isoelectric point**	**One-way ANOVA [unadjusted]**	**One-way ANOVA [adjusted]**	**Average fold change**	**p-value [unadjusted]**	**p-value [adjusted]**	**Average fold change**	**p-value [unadjusted]**	**p-value [adjusted]**	**Average fold change**	**p-value [unadjusted]**	**p-value [adjusted]**
60 kDa heat shock protein	HSPD1	P10809	5.34	0.003700	0.011500	1.05	0.067400	0.098854	1.14	0.001229	0.004158	0.93	0.039915	0.092435
78 kDa glucose-regulated protein	HSPA5	P11021	5.00	0.007480	0.018600	1.13	0.038829	0.063276	1.27	0.003044	0.007441	0.88	0.116920	0.215470
78 kDa glucose-regulated protein	HSPA5	P11021	5.07	0.000767	0.006000	1.06	0.024083	0.042386	1.14	0.000293	0.002148	0.93	0.030787	0.079684
78 kDa glucose-regulated protein	HSPA5	P11021	5.06	0.000214	0.004660	1.13	0.000360	0.003172	1.17	0.001137	0.004158	0.97	0.512009	0.577651
Actin	ACTB	P60709	5.79	0.001430	0.006100	1.18	0.151055	0.189898	0.51	0.015110	0.023332	2.31	0.000238	0.010472
Albumin	ALB	P02768	5.80	0.018300	0.031200	0.94	0.296373	0.318059	0.79	0.011837	0.020032	1.20	0.058425	0.128535
Albumin	ALB	P02768	5.88	0.000823	0.006000	0.93	0.165948	0.202826	0.74	0.000154	0.001359	1.26	0.002706	0.021971
Albumin	ALB	P02768	6.00	0.001060	0.006000	0.88	0.033324	0.056395	0.78	0.000495	0.003115	1.15	0.035448	0.086651
Albumin	ALB	P02768	5.96	0.000380	0.004730	0.91	0.071570	0.101583	0.74	0.000051	0.001133	1.24	0.002886	0.021971
Apolipoprotein A-I	APOA1	P02647	5.33	0.037000	0.046700	0.86	0.003393	0.011658	0.90	0.139776	0.161845	0.96	0.438298	0.561718
ATP synthase subunit beta	ATP5B	P06576	5.07	0.035200	0.045700	1.09	0.672376	0.688013	1.11	0.018637	0.027335	0.98	0.029898	0.079684
Bridgin integrator 2	BIN2	Q9UBW5	5.90	0.000138	0.004660	0.86	0.292033	0.318059	1.29	0.041152	0.058409	0.67	0.002996	0.021971
Calreticulin	CALR	P27797	4.29	0.000057	0.004660	1.10	0.000210	0.003074	1.15	0.000151	0.001359	0.96	0.266406	0.404202
Calreticulin	CALR	P27797	4.29	0.001310	0.006000	1.10	0.003853	0.011790	1.16	0.001100	0.004158	0.94	0.226899	0.369761
Chloride intracellular channel protein 1	CLIC1	O00299	5.32	0.003360	0.011500	0.60	0.200754	0.226491	0.23	0.000768	0.004014	2.67	0.008681	0.031830
Fermitin family homolog 3	FERMT3	Q86UX7	5.88	0.004590	0.013500	0.90	0.009305	0.018610	1.07	0.447489	0.468798	0.76	0.004215	0.026494
Fibrinogen beta chain	FGB	P02675	6.47	0.003570	0.011500	1.21	0.002842	0.011658	1.01	0.870649	0.870649	1.19	0.008621	0.031830
Fibrinogen beta chain	FGB	P02675	6.45	0.021800	0.033300	1.31	0.004326	0.011790	1.29	0.134413	0.161845	1.02	0.422750	0.561718
Fibrinogen beta chain	FGB	P02675	6.70	0.026100	0.037200	1.27	0.005865	0.013583	1.30	0.137674	0.161845	0.98	0.509137	0.577651
Fibrinogen beta chain	FGB	P02675	5.68	0.004670	0.013500	1.09	0.014010	0.026801	1.13	0.008581	0.015102	0.97	0.403286	0.561718

Heat shock protein HSP 90-alpha	HSP90AA1	P07900	5.10	0.001290	0.006000	1.10	0.000333	0.003172	1.06	0.101169	0.130924	1.04	0.225539	0.369761
Integrin alpha-6	ITGA6	P23229	5.05	0.001470	0.006100	0.88	0.006360	0.013992	0.81	0.000912	0.004014	1.10	0.122257	0.215470
Integrin alpha-6	ITGA6	P23229	5.09	0.009920	0.021100	0.89	0.004614	0.011790	0.85	0.003881	0.008132	1.06	0.377881	0.554226
Integrin alpha-6	ITGA6	P23229	5.13	0.002060	0.008100	0.89	0.004450	0.011790	0.84	0.001544	0.004530	1.07	0.249628	0.392273
Integrin alpha-6	ITGA6	P23229	5.12	0.005320	0.014900	0.89	0.003308	0.011658	0.88	0.015378	0.023332	1.01	0.816584	0.855469
Leukocyte elastase inhibitor	SERPINB1	P30740	6.10	0.014000	0.025700	0.90	0.007958	0.016674	0.94	0.152737	0.168011	0.97	0.526556	0.579212
Microtubule-associated protein RP/EB family member 2	MAPRE2	Q15555	5.39	0.033600	0.045000	1.15	0.102117	0.136156	0.92	0.233161	0.250222	1.26	0.010451	0.032846
Microtubule-associated protein RP/EB family member 2	MAPRE2	Q15555	5.41	0.021600	0.033300	1.10	0.185761	0.215092	0.81	0.096493	0.128658	1.35	0.005156	0.028358
Myosin 9	MYH9	P35579	5.33	0.000584	0.006000	0.82	0.052255	0.082115	0.63	0.000152	0.001359	1.32	0.009952	0.032846
Myosin 9	MYH9	P35579	5.33	0.008660	0.019300	0.78	0.058190	0.088288	0.63	0.002497	0.006463	1.25	0.071113	0.148999
Myosin regulatory light polypeptide 9	MYL9	P24844	4.72	0.013500	0.025700	1.09	0.171331	0.203745	1.23	0.012816	0.020885	0.88	0.113500	0.215470
Nucleosome assembly protein 1-like 1	NAP1L1	P55209	4.67	0.007260	0.018600	1.02	0.501883	0.525782	1.23	0.000849	0.004014	0.83	0.002494	0.021971
Proteasome activator complex subunit 1	PSME1	Q06323	5.81	0.000304	0.004730	1.17	0.000089	0.001949	1.15	0.006748	0.012909	1.02	0.740964	0.795181
Protein disulfide-isomerase A1	P4HB	P07237	4.77	0.000004	0.004660	1.11	0.001945	0.010699	1.24	0.000003	0.000136	0.90	0.007801	0.031830
Protein disulfide-isomerase A1	P4HB	P07237	4.79	0.001170	0.006000	1.10	0.001415	0.008894	1.14	0.002481	0.006463	0.96	0.446821	0.561718
Protein disulfide-isomerase A1	P4HB	P07237	4.78	0.002140	0.008100	1.08	0.002351	0.011491	1.11	0.003580	0.008132	0.98	0.499015	0.577651
Protein disulfide-isomerase A6	PDIA6	Q15084	5.12	0.000638	0.006000	1.07	0.001069	0.007837	1.13	0.001377	0.004328	1.00	0.420819	0.561718
Ras-related protein Rab-27B	RAB27B	O00194	5.00	0.000992	0.006000	0.91	0.133365	0.172590	1.12	0.143911	0.162361	0.81	0.006314	0.030868
Translationally-controlled tumor protein	TPT1	P13693	4.84	0.024900	0.036100	1.08	0.101030	0.136156	1.17	0.008013	0.014690	0.93	0.122426	0.215470
Tropomyosin alpha-3 chain	TPM3	P06753	4.75	0.036100	0.046200	0.95	0.697871	0.697871	1.28	0.006184	0.012369	0.74	0.001367	0.021971
Vinculin	VCL	P18206	6.00	0.014200	0.025700	1.25	0.004823	0.011790	1.32	0.051638	0.071002	0.95	0.963131	0.963131
Vinculin	VCL	P18206	6.10	0.000164	0.004660	1.37	0.000060	0.001949	1.45	0.003776	0.008132	0.94	0.884945	0.905525
Vinculin	VCL	P18206	6.20	0.014600	0.025900	1.26	0.003444	0.011658	1.23	0.122439	0.153923	1.03	0.485139	0.577651
Vinculin	VCL	P18206	5.63	0.015300	0.026700	0.75	0.022375	0.041020	1.03	0.702856	0.719202	0.73	0.024526	0.071943

Abbreviations: LA – lupus anticoagulant; TE – thromboembolism;

Supplementary Table 2: Altered platelet protein abundances between LA-positive patients receiving antithrombotic agents and LA-positive patients without antithrombotic agents.

		Antithrombotic agent (n=28)/ No antithrombotic agent (n=19)		
**Protein name**	**Gene name**	**Average fold change**	**p-value [unadjusted]**	**p-value [adjusted for thrombosis]**
Microtubule-associated protein RP/EB family member 2	MAPRE2	0.62	0.007	0.211
Vinculin	VCL	1.05	0.021	0.426
Fibrinogen beta chain	FGB	0.99	0.024	0.787
Nucleosome assembly protein 1-like 1	NAP1L1	1.07	0.033	0.281
Actin	ACTB	1.07	0.043	0.300
60 kDa heat shock protein	HSPD1	0.93	0.049	0.215

Abbreviations: LA – lupus anticoagulant

Supplementary Table 3: Altered platelet protein abundances between LA-positive patients with a history of venous TE and LA-positive patients with a history of arterial TE analyzed by 2D-DIGE.

		LA+VTE+ (n=22)/ LA+ATE+ (n=7)	
**Protein name**	**Gene name**	**Average fold change**	**p-value [unadjusted]**
Apolipoprotein A-I	APOA1	0.67	0.0003
Bridgin integrator 2	BIN2	1.44	0.0031
Myosin regulatory light polypeptide 9	MYL9	1.30	0.0154
Tropomyosin alpha-3 chain	TPM3	1.26	0.0169
Leukocyte elastase inhibitor	SERPINB1	0.90	0.0386

Abbreviations: LA – lupus anticoagulant; TE – thromboembolism; ATE – arterial thromboembolism; VTE – venous thromboembolism

Supplementary Figures:

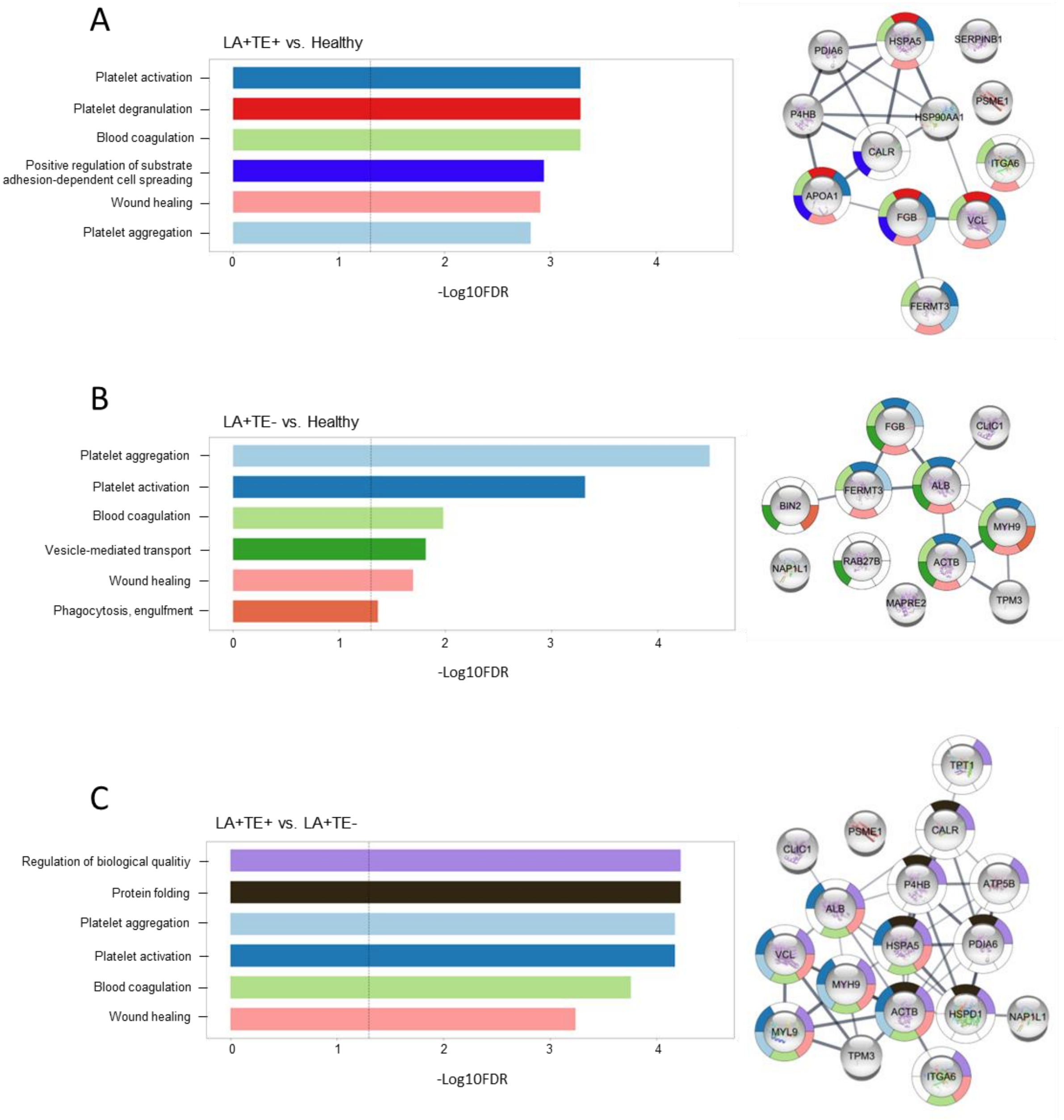

Supplementary Fig. 1: STRING pathway analysis of altered platelet proteins between (A) LA+TE+ patients vs. healthy controls, (B) LA+TE- patients vs. healthy controls and (C) LA+TE+ patients vs. LA+TE- patients. The network view summarizes the network of predicted associations for the proteins (nodes) differentially expressed in each comparison. The edges represent the predicted functional associations. The thickness of the edge indicates the degree of confidence prediction of the interaction. The enrichment graphs depict the 6 most significantly enriched GO Biological Processes terms (FDR < 0.05, dashed line) for each comparison studied. Each term is represented by a specific color, and the nodes annotated with that term show the respective color. Abbreviations: LA – lupus anticoagulant; TE – thromboembolism; FDR – false discovery rate

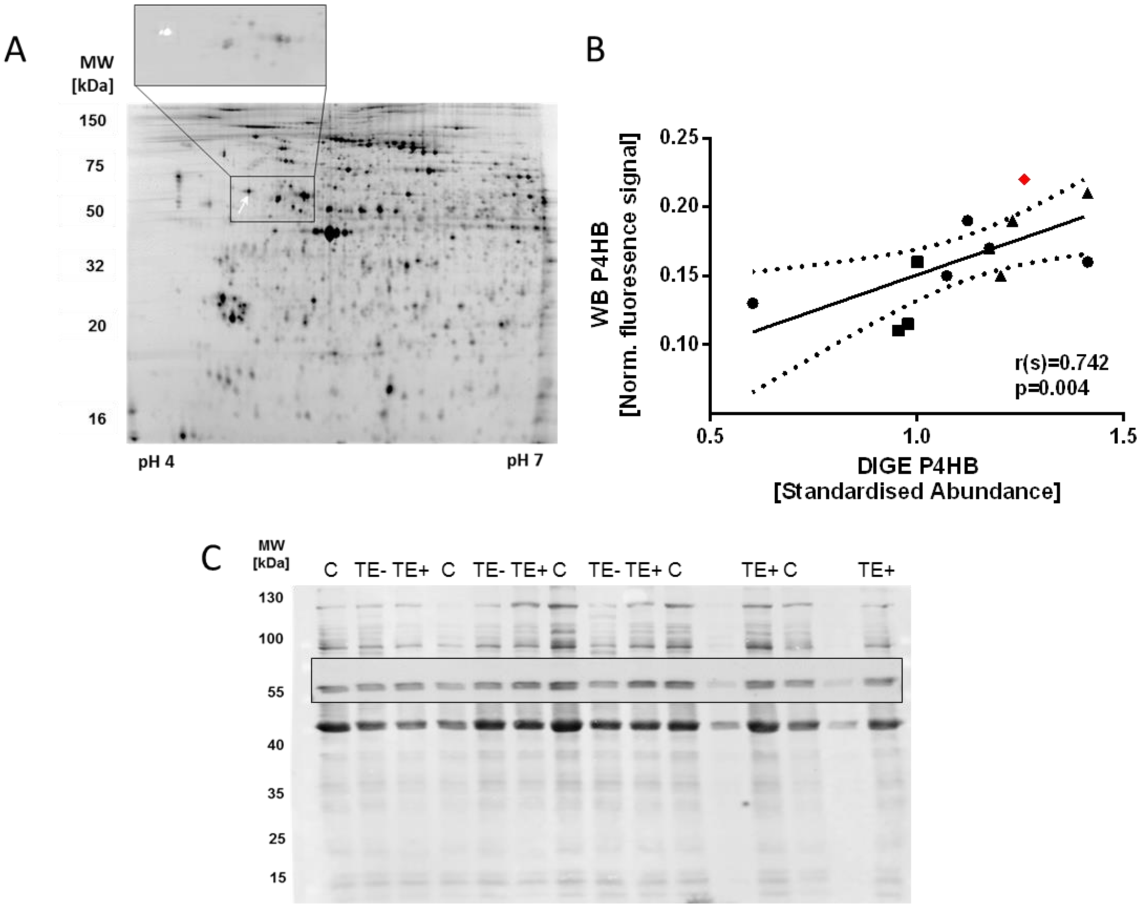

Supplementary Fig. 2: Two-dimensional and quantitative 1-D WB analysis of P4HB. (A) Representative 2-D WB image of platelet P4HB probed with monoclonal protein disulfide-isomerase A1 (P4HB), clone RL90 antibody. 36 µg Cy2-labeled protein was applied to IEF on a 24 cm pH 4-7 IPG strip. Proteins were separated by a 11.5% SDS-PAGE according to their molecular weight. Antibody fluorescence signals were detected with a Typhoon FLA 9500 imager (Protein of interest is highlighted with a white arrow). The cut-out region shows an overlay from Cy2-labeled whole protein stain and specific WB signal, where immune-identified proteins are displayed as white spots. (B) Scatter blot and correlation analysis of P4HB protein levels measured by 2D DIGE and qWB analysis (Spearman's rank correlation coefficient). (C) Representative 1-D WB image from P4HB. Twelve µg of platelet proteins from LA+TE+ patients (n=5), LA+TE- patients (=3) and healthy controls (n=5) were applied per lane and separated by a 11.5% SDS-PAGE. The specific pan-P4HB antibody recognized one protein band with molecular weight of about 58 kDa, well corresponding to the positions of the P4HB spot found by 2D-DIGE analysis. Ruthenium-based whole-protein stain of blotted proteins was performed to assess equal protein application. The image section shows the whole-protein stain and the overlay of the antibody signals detected by fluorescence signal (Cy5). Healthy controls [C], LA+ TE- patients without TE [TE-] and LA+ patients with TE [TE+]; Abbreviations: WB – western blot; 2D-DIGE – two-dimensional differential in-gel electrophoresis; IEF – isoelectric focusing; MW – molecular weight; kDa – kilodalton; TE – thromboembolism; LA – lupus anticoagulant

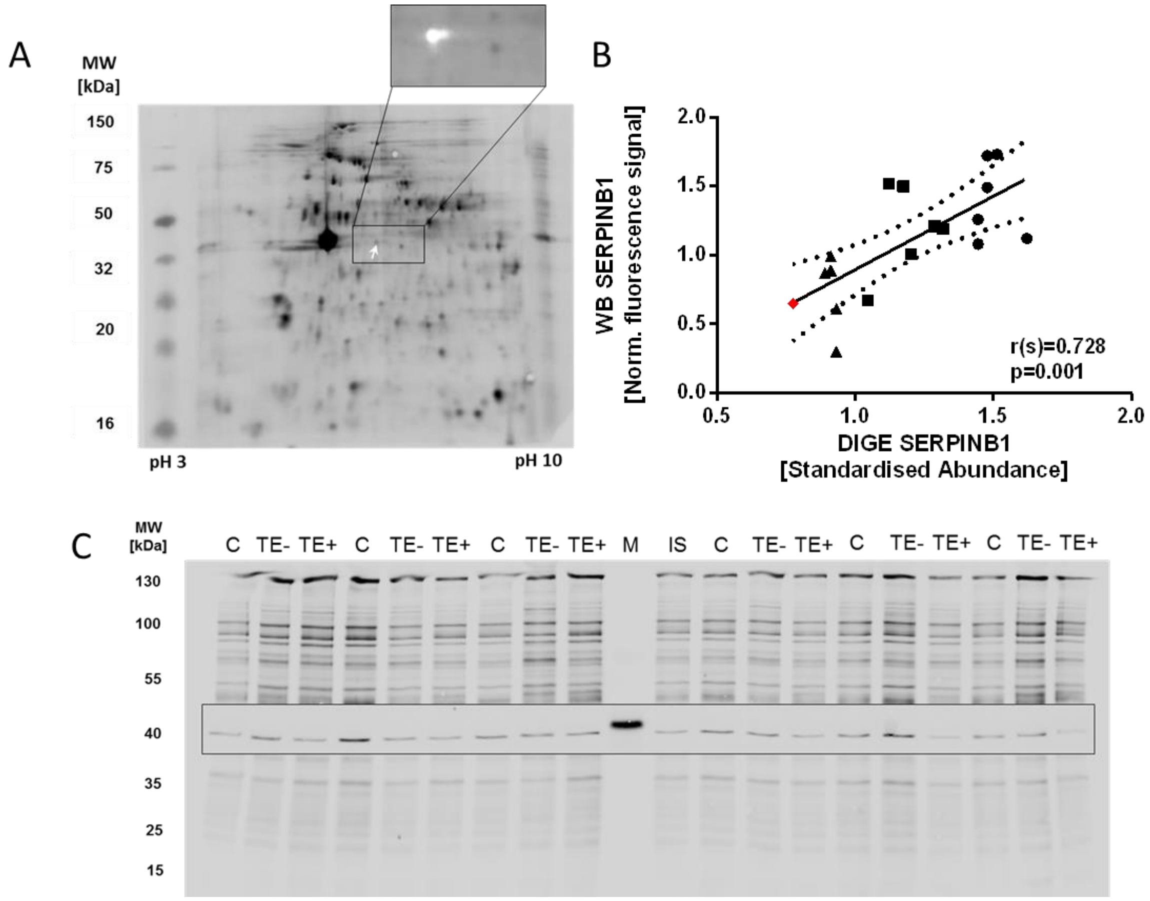

Supplementary Fig. 3: Two-dimensional and quantitative 1-D WB analysis of SERPINB1. (A) Representative 2-D WB image of platelet SERPINB1 probed with monoclonal leukocyte elastase inhibitor antibody clone EPR13305(B) antibody. 36 µg Cy2-labeled protein were applied to IEF on a 12 cm pH 3–10 IPG strip. Proteins were separated by a 11.5% SDS-PAGE according to their molecular weight. Antibody fluorescence signals were detected with a Typhoon FLA 9500 imager (Protein of interest is highlighted with a white arrow). The cut-out region shows an overlay from Cy2-labeled whole protein stain and specific WB signal, where immune-identified proteins are displayed as white spots. (B) Scatter blot and correlation analysis of Scatter blot and correlation analysis of SERPINB1 protein levels measured by 2D DIGE and qWB analysis (Spearman's rank correlation coefficient). (C) Representative 1-D WB image from SERPINB1. Twelve µg of platelet proteins from LA+TE+ patients (n=6), LA+TE- patients (=6) and healthy controls (n=6) were applied per lane and separated by a 11.5% SDS-PAGE. The specific pan-SERPINB1 antibody recognized one protein band with molecular weight of about 43 kDa, well corresponding to the positions of the SERPINB1 spot found by 2D-DIGE analysis. Ruthenium-based whole-protein stain of blotted proteins was performed to assess equal protein application. The image section shows the whole-protein stain and the overlay of the antibody signals detected by fluorescence signal (Cy5). Healthy controls [C], LA+ TE- patients without TE [TE-] and LA+ patients with TE [TE+]; M – marker, IS – internal standard for standardization. Abbreviations: WB – western blot; 2D-DIGE – two-dimensional differential in-gel electrophoresis; IEF – isoelectric focusing, MW – molecular weight; kDa – kilodalton; TE – thromboembolism; LA – lupus anticoagulant
